# Stochastic Collisional Quantum Thermometry

**DOI:** 10.3390/e23121634

**Published:** 2021-12-06

**Authors:** Eoin O’Connor, Bassano Vacchini, Steve Campbell

**Affiliations:** 1 School of Physics, University College Dublin, Belfield, D04 V1W8 Dublin, Ireland; eoin.oconnor2@ucdconnect.ie; 2 Centre for Quantum Engineering, Science, and Technology, University College Dublin, Belfield, D04 V1W8 Dublin, Ireland; 3 Dipartimento di Fisica “Aldo Pontremoli”, Università degli Studi di Milano, Via Celoria 16, 20133 Milan, Italy; bassano.vacchini@mi.infn.it; 4 Istituto Nazionale di Fisica Nucleare, Sezione di Milano, Via Celoria 16, 20133 Milan, Italy

**Keywords:** open quantum systems, quantum thermometry, collision models

## Abstract

We extend collisional quantum thermometry schemes to allow for stochasticity in the waiting time between successive collisions. We establish that introducing randomness through a suitable waiting time distribution, the Weibull distribution, allows us to significantly extend the parameter range for which an advantage over the thermal Fisher information is attained. These results are explicitly demonstrated for dephasing interactions and also hold for partial swap interactions. Furthermore, we show that the optimal measurements can be performed locally, thus implying that genuine quantum correlations do not play a role in achieving this advantage. We explicitly confirm this by examining the correlation properties for the deterministic collisional model.

## 1. Introduction

Accurately determining the temperature of a physical system is a ubiquitous task. For quantum systems, measuring the temperature becomes a significantly more involved job, in part due to the inherent fragility of quantum states, and, more pointedly, due to the fact that temperature itself is not a quantum observable. Recently, significant advances in thermometry schemes for quantum systems have been proposed [1,2,3,4,5,6] (see Ref. [7] for an extensive review). The thermometric precision of a probe in equilibrium with the sample is limited by the thermal Cramer–Rao bound, which is inversely proportional to the heat capacity, *C*, of the thermometer: ${\left(\Delta T/T\right)}^{2}\geq k_{B}/NC$. However, quantum systems have the additional freedom to exploit resources, such as entanglement [8,9] and coherence [10,11,12], to gain an advantage over their classical counterpart [13,14,15,16,17,18,19,20,21]. By making use of these resources, along with collective measurements on multiple probes, it is possible to surpass the 1/N scaling of the Cramer–Rao bound.

A typical “direct” thermometry scheme will involve a number of probes coupled to the system of interest (environment) and, after a suitable interaction, these probes are measured in order to estimate the temperature. However, recently, an alternative approach was proposed that exploits collision models [22,23,24,25,26,27,28,29,30,31,32] that involve a stream of auxiliary systems (often termed “ancillae”) interacting with an intermediary system, which is directly coupled to the environment [33]. The collisions occur for a sufficiently small time, such that the auxiliary systems never fully thermalise with the environment–intermediary system compound. However, information about the temperature of the environment is indirectly imprinted onto the auxiliaries, and thus this scheme allows us to make use of this additional out-of-equilibrium information to enhance the precision of the temperature estimation [33,34,35].

In this paper, we extend this collisional approach to allow for stochasticity in the waiting times between the collisions. We show that introducing this stocasticity leads to a broadening of the range of parameters in which a meaningful advantage is gained over a direct thermometry scheme, where *N*-probes thermalise with the environment and are then measured. Interestingly, for a dephasing interaction between the intermediary system and auxiliaries, we establish that the correlations generated in the collisional thermometry approach are purely classical and, therefore, the demonstrated advantage can be gained by performing simple local measurements.

The remainder of the paper is organised as follows. Section 2 gives an outline of the various topics and techniques employed in collisional quantum thermometry. These techniques are then examined more closely to determine the exact role of correlations and the free parameters. In Section 3, we introduce stochasticity at the level of the waiting time between collisions. We analyse how this stochasticity affects the precision of the measurements and the form of the optimal measurements. We also discuss how our results extend to different forms of measurements. Finally, our conclusions and some further discussions are presented in Section 4.

## 2. Quantum Thermometry

### 2.1. Thermal Fisher Information

The maximum precision with which the temperature of the environment can be measured is determined by the quantum Cramer–Rao bound

(1) $${\left(\Delta T\right)}^{2}\geq \frac{1}{F(T,\rho )},$$

where F(T,ρ) is the quantum Fisher information (QFI) of the state, ρ, at temperature *T*. In order to gain information from ρ, a measurement, or positive operator valued measure (POVM), Π, must be performed on the state. The outcome of this measurement is then determined by the probability distribution $p\left(x\right)\;=\;\mathrm{tr}(\Pi_{x}\rho )$, where $\Pi_{x}$ is the POVM element associated with measurement outcome *x*. The Fisher information associated with this measurement is given by

(2) $$F\left(T,\Pi ,\rho \right)=\sum_{x}p\left(x\right){\left(\frac{\partial }{\partial T}\ln p\left(x\right)\right)}^{2}.$$

The QFI is attained by maximising this Fisher information over all POVMs. This maximisation can be determined from the expectation value of the square of the symmetric logarithmic derivative, $F\left(T,\rho \right)\;=\;\mathrm{tr}\left\{\rho \Lambda^{2}\right\}$, with Λ defined implicitly by $2\partial_{t}\rho \;=\;\Lambda \rho +\rho \Lambda$.

For a typical thermometry scheme involving a number of probes fully thermalising with the environment, the corresponding QFI is known as the thermal Fisher information, and is given by

(3) $$F\left(T,\rho \right)=F_{th}=\frac{C}{k_{B}T^{2}},\;\;C=\frac{\langle H_{p}^{2}\rangle -{\langle H_{p}\rangle }^{2}}{k_{B}T^{2}}$$

where *C* is the heat capacity of the probe, $H_{p}$ is the probe Hamiltonian, and the probes are assumed to have reached the Gibbs state with inverse temperature $\beta \;=\;1/k_{B}T$. In the case of a qubit probe with frequency Ω, the thermal Fisher information is

(4) $$F_{th}=\frac{1}{\bar{n}\left(\bar{n}+1\right)\left(2\bar{n}+1\right)}{\left(\frac{\partial \bar{n}}{\partial T}\right)}^{2}$$

where

(5) $$\bar{n}\;=\;1/\left(e^{\hbar \Omega /k_{B}T}-1\right),$$

is the mean occupation number at frequency Ω and temperature *T*. Thus, estimating $\bar{n}$ is equivalent to estimating temperature *T*. Equation (4) provides a lower bound, which we can benchmark the performance of our stochastic collision scheme against.

### 2.2. Collisional Thermometry

Here, we recap the basic ingredients of the collisional thermometry scheme outlined in Refs. [33,34]. Our set up consists of a (large) environment, *E*, at fixed temperature *T*, and it is this temperature that we wish to estimate. The environment is coupled to an intermediary system, *S*, such that, in the absence of any other interaction, *S* will reach thermal equilibrium with *E*. This intermediary system is, in turn, coupled to a stream of independent and identically prepared auxiliary units, $A_{i}$, which form the collisional bath. In what follows, we will assume both *S* and all $A_{i}$’s are qubits and that the *S*-$A_{i}$ interaction is unitary. Information about the temperature is then gained by performing measurements on the $A_{i}$’s, either individually or in batches. This is in contrast to standard probe-based thermometry, where the probes interact directly with the environment, with the best precision occurring when they are permitted to thermalise fully before being measured. We assume that the *S*-$A_{i}$ interaction time, $\tau_{SA}$, is small compared to the system–environment coupling time, $\tau_{SE}$, allowing us to neglect the system–environment coupling during the collisions. After *N* collisions, the system and auxiliaries are given by the combined state

(6) $$\rho_{S,A_{1},\ldots ,A_{N}}=U_{SA_{N}}\circ E\circ U_{SA_{N-1}}\circ \ldots \circ E\circ U_{SA_{1}}\left(\rho_{S}⊗\rho_{A_{1}}⊗\ldots ⊗\rho_{A_{N}}\right)$$

where $U_{SA_{i}}\left(\circ \right)\;=\;U_{SA_{i}}\circ U_{SA_{i}}^{†}$ and E corresponds to the map induced by the *S*-*E* interaction acting on the intermediary system in between the collisions.

We model the *S*-*E* interaction by a general thermalising master equation in the weak coupling limit, which, in the interaction picture, is given by

(7) $$\frac{d\rho_{S}}{dt}=L\left(\rho_{S}\right)=\gamma \left(\bar{n}+1\right)D\left[\sigma_{-}^{S}\right]\left(\rho_{S}\right)+\gamma \bar{n}D\left[\sigma_{+}^{S}\right]\left(\rho_{S}\right)$$

where $D\left[A\right]\left(\rho \right)\;=\;A\rho A^{†}-\frac{1}{2}\left\{A^{†}A,\rho \right\}$, and γ is the system–environment coupling constant. We can now calculate E in Equation (6) by integrating Equation (7) over the time between subsequent collisions $\tau_{SE}$. The resulting channel takes the form $E\;=\;e^{\tau_{SE}L}$. This is a thermalising map that brings *S* towards the Gibbs state, $\rho_{S}^{th}$, i.e., $E\left(\rho_{S}^{th}\right)\;=\;\rho_{S}^{th}$. We choose the intermediary system and auxiliaries to be resonant, i.e., $H_{S}\;=\;H_{A}\;=\;\hbar \Omega \sigma_{z}/2$. The system, *S*, therefore experiences the stroboscopic map

(8) $$\rho_{S}^{i}={\mathrm{tr}}_{A_{i}}\left\{U_{SA_{i}}\circ E\left(\rho_{S}^{i-1}⊗\rho_{A_{i}}\right)\right\}:=\Phi \left(\rho_{S}^{i-1}\right).$$

For equally spaced collision times, this map has a unique steady state $\rho_{S}^{*}\;=\;\Phi \left(\rho_{S}^{*}\right)$, which is not necessarily the Gibbs state, with the notable exception of a pure dephasing interaction between *S* and $A_{i}$, as outlined in the following section.

### 2.3. Dephasing Interactions

We begin by focusing on a *ZZ* interaction between the collisional bath and the system,

(9) $$H_{SA_{i}}^{ZZ}=\frac{\hbar g}{2}\sigma_{S}^{Z}\sigma_{A_{i}}^{Z},$$

which leads to a dephasing in the energy eigenbasis and is also referred to as an indirect measurement interaction. We tune the effective system–environment coupling, $\gamma \tau_{SE}$, and the effective *S*-$A_{i}$ coupling, $g\tau_{SA}$. Due to the fact that Equation (9) only affects the off-diagonal elements (i.e., coherences), and assuming that *S* begins in thermal equilibrium with *E* before any interactions with the auxiliaries occur, the reduced state of *S* will remain in the Gibbs state. However, provided that a suitable choice of initial state for the auxiliaries is chosen, information about the environment temperature can be imparted to the collisional bath. For a single auxiliary unit, the QFI is maximised when its initial state is perpendicular to the *Z*-axis, e.g., $|{+}_{x}\rangle \;=\;\left(|g\rangle +|e\rangle \right)/\sqrt{2}$, with the corresponding QFI given by

(10) $$F^{|{+}_{x}\rangle }=\frac{1-\cos(2g\tau_{SA})}{2}F^{th},$$

which is clearly maximised when the coupling parameter $g\tau_{SA}\;=\;\pi /2$. However, from Equation (10), we clearly see that, for the considered interaction, it is impossible to beat the thermal Cramer–Rao bound by measuring a single auxiliary unit. When multiple $A_{i}$’s collide with the system in succession, correlations can be established between them; however, for long times between successive collisions, the (re)thermalisation due to the system–environment interaction will destroy all correlations, leaving the total QFI equal to the sum of all of the individual QFIs for each $A_{i}$, i.e., *N* times Equation (10). Conversely, in the short inter-collision time limit, the first collision provides as much information about the temperature as possible and, while classical correlations are established between successive $A_{i}$’s, no additional information can be gained about the temperature of the environment by measuring multiple auxiliary units. Between these two extremes, relevant information about the temperature of the environment can be encoded into the auxiliary systems and provide significant advantages in precision over the thermal Cramer–Rao bound.

**Figure 1:**
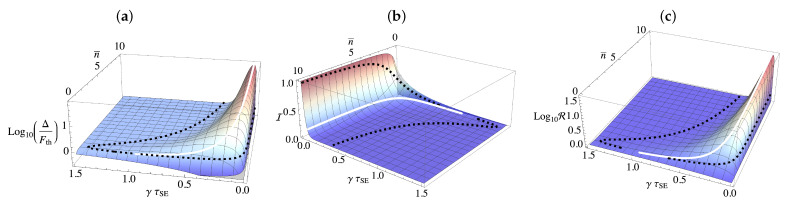
(**a**) Plot of the (log of the) ratio between Δ, Equation (12), and $F_{th}$. Positive regions indicate parameter regimes where a thermometic advantage is achievable via the collisional scheme. (**b**) Mutual information between two adjacent auxiliary units after each has interacted with the system via a ZZ interaction for a deterministic collisional therometry protocol. (**c**) Measure of the interdependence between γ and $\bar{n}$ captured by Equation (15) for a deterministic protocol with N=2 (arbitrary choice). In all panels, the area captured by the dashed black line represents the region in parameter space where the scheme achieves an advantage over the thermal QFI. The white line corresponds to the value of $\bar{n}$ where the QFI is maximal.

Fixing the optimal *S*-$A_{i}$ collision, such that $g\tau_{SA}\;=\;\pi /2$, the QFI for *N* auxiliaries interacting with *S* is given by [34]

(11) $$\begin{array}{cc} \; & F_{N}^{|{+}_{x}\rangle }=F^{th}+\left(N-1\right)\Delta ,\;\mathrm{with}, \end{array}$$

(12) $$\begin{array}{cc} \Delta & =\frac{{\left(1+\bar{n}\right)}^{2}\left[1-e^{\Gamma }\left(1+2\bar{n}\Gamma \right)\right]}{\left(1-e^{\Gamma }\right)\left(1-\frac{\left(1-e^{\Gamma }\right)\bar{n}}{1+2\bar{n}}\right)}+\frac{{\bar{n}}^{2}\left[-1+e^{\Gamma }\left(1-2\left(1+\bar{n}\right)\Gamma \right)\right]}{\left(1-e^{\Gamma }\right)\left(1-\frac{\left(1-e^{\Gamma }\right)\left(1+\bar{n}\right)}{1+2\bar{n}}\right)} \end{array}$$

where $\Gamma \;=\;\gamma \left(2\bar{n}+1\right)\tau_{SE}$ is the effective thermalisation rate of the system. From Equation (11), we find that the condition for beating the thermal Cramer–Rao bound corresponds to $\Delta /F_{th}\;>\;1$, shown in Figure 1a [34]. Furthermore, the expression for Δ demonstrates that knowledge of the *S*-*E* coupling parameter, $\gamma \tau_{SE}$, is essential to achieve any boost in thermometric performance. For the remainder of this section, we will assume a deterministic collisional scheme, namely, the system and the environment interaction time is identical between each of the collisional events, i.e., $\gamma \tau_{SE}$ is the same between each collision. Thus, we consider the same setting as Refs. [33,34] of equally distributed collisions, and in Section 3, we introduce stochasticity.

### 2.4. Role of Correlations

Given that, for the ZZ interaction, measuring a single auxiliary cannot outperform the thermal Cramer–Rao bound, it is natural to ask what allows for the enhancement when multiple units are measured and how this relates to the correlations established between successive $A_{i}$’s and/or between *S* and a given auxiliary. We can quantify these correlations via the bipartite mutual information

(13) $$I=S\left(\rho_{A}\right)+S\left(\rho_{B}\right)-S\left(\rho_{AB}\right),$$

where S(·) is the von Neumann entropy. This quantity captures all correlations, both quantum and classical, present in the state. In Figure 1b, we show the mutual information shared between two successive auxiliaries, i.e., $\rho_{A_{i}A_{i+1}}$, where it clearly appears that significant correlations are established that depend on the time between each collision and the temperature of the environment. The dashed black line encloses the area in which an advantage of >1% can be gained from this setup and indicates that, while there appears to be a qualitative relationship between the magnitude of the mutual information shared between the auxiliaries and the corresponding thermometric performance, with some amount of mutual information clearly being necessary in order to gain an advantage, remarkably, too much correlation actually results in the QFI being lower than the thermal Fisher information. The boundary is delineated by the white line, which tracks the peak QFI for each value of $\bar{n}$. We can further characterise the type of correlations present by determining the quantum discord [36,37], which captures the genuine quantum nature of the correlations present, and, in this case, turns out to be identically zero. This implies that the correlations contributing to the increased metrological performance are purely classical.

### 2.5. Parameter Dependence

We see from Equation (12) that the advantage gained from this collisional approach depends on the effective thermal relaxation parameter $\Gamma \;=\;\gamma \left(2\bar{n}+1\right)\tau_{SE}$. Consequently, in order to maximise this advantage both γ and $\tau_{SE}$ must be known with certainty. While $\tau_{SE}$ corresponds to the time between collisions, which, given sufficient control over the collisional bath, can, in principle, be known, the parameter γ, which corresponds to the coupling strength between the system and the environment, is more delicate. In certain circumstances, it may be that, prior to any measurements, γ is known for the setup. However, when this is not the case, or if the bath is prone to some other disturbance, determining it precisely is essential [38].

We can demonstrate the importance of knowing γ through the total variance of a measurement in multi-parameter estimation by summing the variances of all parameters. When estimating *m* unknown parameters, we obtain the following chain of inequalities [39].

(14) $$\sum_{a}^{m}\mathrm{var}\left(x_{a}\right)\geq \frac{1}{m}\mathrm{tr}\left\{F^{-1}\right\}\geq \sum_{a}\frac{1}{mF_{aa}},$$

where F is the QFI matrix. In our case, m=2 and $x_{a}\;\in \;\left\{\bar{n},\gamma \right\}$. The second inequality is only saturated when all of the parameters are independent of each other, and, therefore, comparing the ratio between the second and third terms, which we denote as

(15) $$R=\frac{\mathrm{tr}\left\{F^{-1}\right\}}{\sum_{a}\frac{1}{F_{aa}}},$$

allows us to identify the areas in which knowledge of γ is necessary in order to estimate the temperature. Figure 1c shows the peaks of this ratio line up perfectly with the peaks of the QFI. Additionally, if one has no knowledge of γ, it is impossible to gain any advantage over the thermal Fisher information. In fact, the QFI is smaller than the thermal Fisher information in the case when the time between collisions is small.

## 3. Stochastic Approach

The previous section outlined the basic ingredients of the collisional thermometry scheme for the deterministic case introduced in Refs. [33,34]. We now turn our attention to our main focus: introducing stochasticity at the level of the time between collisions, $\tau_{SE}$, while keeping the average collision time, $\tau_{SA}$, consistent with the previous section.

### 3.1. Random Collision Times

In nature, interactions will not generally occur in fixed intervals or at deterministic times. Rather, processes are typically random, with the time between interactions captured by a suitable probability distribution, the waiting time distribution (WTD). Within the framework of open quantum systems, collision models allow us to introduce such randomness, either in the intervals between successive collisions or in the collision time itself, and are referred to as stochastic collision models [40,41,42].

**Figure 2:**
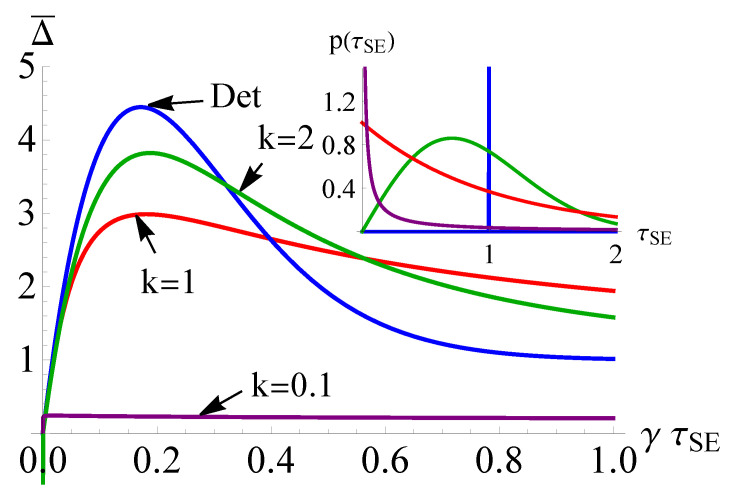
Comparison of the value of the quantum Fisher information for various Weibull distributions of the collision time interval (see Equation (16)), with the deterministic case, for $\bar{n}\;=\;2$. Similar behavior is seen for other values of temperature above $\bar{n}\;=\;1.5$. *Inset:* Distributions for various values of *k* shown in the main panel.

While employing WTDs in this sense clearly brings collision models closer to modelling real physical systems [43,44,45], here, we explore how introducing such randomness affects the performance of the collisional thermometry scheme. As we shall demonstrate, stochastic collision models allow us to achieve a greater range of parameter estimation over deterministic collision models without significantly sacrificing the maximal achievable precision. For concreteness, we shall focus on the Weibull renewal distribution,

(16) $$p\left(t\right)=\frac{k}{\lambda }{\left(\frac{t}{\lambda }\right)}^{k-1}e^{-{\left(t/\lambda \right)}^{k}},$$

where λ is the average time between collisions and *k* determines the shape of the distribution, cfr. the inset of Figure 2. In particular, large *k* tends to regular intervals between collisions, i.e., k→∞ corresponds to deterministic, equally-spaced collisions, as considered in Refs. [33,34] and Section 2.4 and Section 2.5, whereas small *k* is characterised by bursts of collisions followed by long breaks [42]. For k=1, the WTD corresponds to the exponential distribution characterising a Poisson point process. We remark that our results remain qualitatively unaffected for other families of WTD, e.g., Erlang distributions.

**Figure 3:**
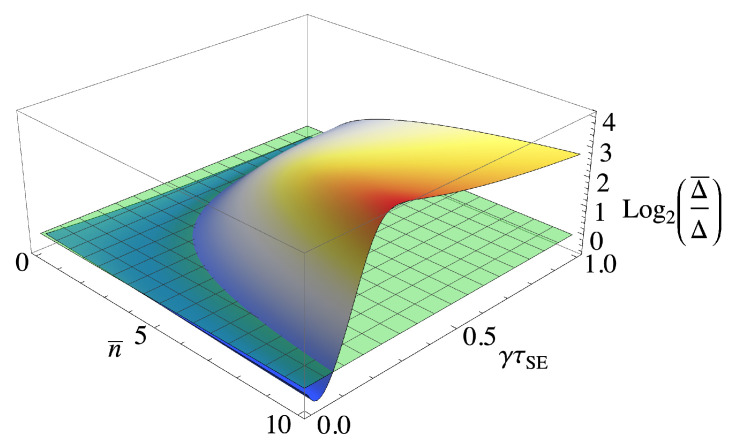
Comparison of the ratio between Δ for a Weibull distribution for the exponential distribution, i.e., k=1, and a deterministic equally spaced waiting time distribution. The green plane represents the crossing point where one term becomes larger than the other. $\gamma \tau_{SE}$ is the average time between collisions.

As we see from Equation (11), when the waiting time between subsequent collisions is deterministic and constant, it is possible to obtain a QFI that is orders of magnitude higher than the thermal Fisher information for specific values of the coupling parameters and temperature [33,34]. However, a drawback of this is that such high precision is restricted to a narrow parameter range, and is delicately dependent on the temperature of the environment. Such a situation is clearly not ideal given that the temperature is the very quantity that we wish to estimate [33,34]. Approaches to address this issue include introducing global estimation schemes [13,46,47,48,49] and biased estimators [35]. Here, we demonstrate that, if the interactions are random and governed by a particular WTD, this randomicity has an important effect on the value of the parameter Δ that determines the possible advantage over the thermal Fisher information. It is straightforward to extend the proof of Equation (12) from Ref. [34] to random waiting time distributions

(17) $$F_{N}^{|{+}_{x}\rangle }=F^{th}+\sum_{i=1}^{N-1}\Delta_{i},$$

where $\Delta_{i}$ takes an identical form to the one given in Equation (12), except with $\tau_{SE}$ now replaced with a variable time $\tau_{SE}^{i}$. To determine the average performance of a particular WTD, p(t), we now average over each collision time

(18) $$F_{N}^{|{+}_{x}\rangle }=\int_{0}^{\infty }\cdots \int_{0}^{\infty }\prod_{i=1}^{N-1}d\tau_{SE}^{i}p\left(\tau_{SE}^{i}\right)F_{N}^{|{+}_{x}\rangle }=F^{th}+\left(N-1\right)\bar{\Delta },$$

where $\bar{\Delta }\;=\;\int_{0}^{\infty }d\tau_{SE}\;p\left(\tau_{SE}\right)\;\Delta$, with the WTD p(t) being any positive function that satisfies $\int_{0}^{\infty }dt\;p\left(t\right)\;=\;1$. In Figure 3, we show the (log of the) ratio between $\bar{\Delta }$ for an exponential distribution, i.e., k=1 (arbitrary choice) and the deterministic Δ. We can see that the randomness allows for a significant performance boost (up to 10 times larger) over a wide range of parameters at the cost of a slight sensitivity loss when the deterministic QFI is maximal.

While we have established that an advantage over the regularly spaced collisions can be achieved for a particular choice of WTD, we now turn our attention to how the particular form of distribution affects the performance. As mentioned previously, varying *k* in the Weibull distribution, Equation (16), interpolates between distributions with regularly space collisions for k→∞ to collisions in batches followed by long pauses as k→0. We compare the QFI for various values of *k* at a fixed (arbitrarily chosen) value of temperature, corresponding to $\bar{n}\;=\;2$, in Figure 2. For larger values of *k*, we find that the behaviour tends to the deterministic case, which is characterised by a QFI with a large peak that is narrow in the parameter range. While the scheme is highly effective, it requires a precise knowledge of the coupling between system and environment. However, for smaller values of *k* leading to a more random sequence of collisions, we find that the range over which an advantage can be demonstrated is significantly broadened, albeit at the expense of reducing the “maximum” achievable precision. Thus, by introducing stochasticity to the process, we are able to alleviate the need for precise knowledge of the optimal system–environment coupling. Interestingly, there is a limit to how small *k* can be and still retain an advantage, with very small *k* leading to collisions occurring so close together that no additional information can be gained.

### 3.2. Optimal Measurements

While the QFI places an asymptotic bound on the accuracy of parameter estimation, it does not provide details on precisely what POVM should be implemented in order to saturate the bound. Therefore, identifying the measurements that must be performed on the auxiliary units is important for assessing the implementability of the scheme, something which is particularly relevant for our stochastic collisional approach, in order to assess whether optimal measurements depend on the waiting time between collisions. To find the optimal measurement, we need the symmetric logarithmic derivative (SLD) operator $L_{a}$ for parameter $x_{a}$. In terms of the eigen-decomposition of $\rho \;=\;\sum_{i}\lambda_{i}|\lambda_{i}\rangle \;\langle \lambda_{i}|$, the SLD operator is [39]

$$\langle \lambda_{i}|L_{a}|\lambda_{j}\rangle =\delta_{ij}\frac{\partial_{a}\lambda_{i}}{\lambda_{i}}+\frac{2(\lambda_{j}-\lambda_{i})}{\lambda_{i}+\lambda_{j}}\langle \lambda_{i}|\partial_{a}\lambda_{j}\rangle .$$

For our scheme, we find that the eigenvectors $\left\{|l_{i}\rangle \right\}$ of $L_{a}$ are independent of $x_{a}$, and that the Fisher information is

$$\begin{array}{cc} I_{aa} & =\sum_{i}\frac{\langle l_{i}|\partial_{a}\rho {|l_{i}\rangle }^{2}}{\langle l_{i}|\rho |l_{i}\rangle }=\sum_{i}\frac{\langle l_{i}|\rho L_{a}+L_{a}\rho {|l_{i}\rangle }^{2}}{\langle l_{i}|\rho |l_{i}\rangle }\\ \; & =\mathrm{tr}\left\{\rho L_{a}^{2}\right\}=F_{aa} \end{array}$$

where $F_{aa}$ is the quantum Fisher information for parameter $x_{a}$. This implies that the optimal measurement corresponds to one performed over the $\left\{|l_{i}\rangle \right\}$ basis. For the ZZ interaction considered with $g\tau_{SA}\;=\;\pi /2$, the eigenvectors $|\lambda_{i}\rangle$ of ρ are independent of *T* and γ, meaning that the optimal measurement is precisely the measurement in the $\left\{|\lambda_{i}\rangle \right\}$ basis, and is the same for both *T* and γ. For the auxiliary units initialised in the $|x_{+}\rangle$ state considered here, there is some ambiguity in the measurement basis due to degeneracy in the eigenvalues. However, the simplest basis is $|y_{i}\rangle \ldots |y_{j}\rangle$ for i,j∈{+,−} and $y_{+}\;=\;\left\{1,i\right\}$, $y_{-}=\left\{1,-i\right\}$, with this result holding for any number of auxiliary units. Thus, the optimal measurements involve only product states, and can therefore be performed using only local, single-qubit projective measurements, which is consistent with the results of Section 2.4, where we established that there are no genuinely quantum correlations present in the state.

### 3.3. Partial Swap Interactions

We conclude our analysis by considering an alternative form for the *S*-$A_{i}$ interaction that has been considered frequently in collisional thermometry [33,34,35]. The partial swap (also referred to as an exchange) interaction is given by

(19) $$H_{SA}^{\mathrm{Swap}}=\hbar g\left(\sigma_{S}^{+}\sigma_{A}^{-}+\sigma_{S}^{-}\sigma_{A}^{+}\right).$$

where, similarly to the previous case, we are able to tune the effective couplings, $\gamma \tau_{SE}$ and $g\tau_{SA}$. In contrast to the ZZ interaction, now the system and auxiliaries will exchange energy as well as coherences, and thus the intermediary system will not remain in the Gibbs state throughout the dynamics. As a consequence, it is possible to gain a significant advantage over the thermal Fisher information from just a single auxiliary unit. When a single collision corresponds to a full swap, the QFI is maximised and there are no correlations established between subsequent collisional units. Clearly, this interaction is highly disruptive to the intermediary system. We now find that the optimal state for each $A_{i}$ is the ground state [34] and that the corresponding optimal measurements are projective measurements in the energy eigenstates. It is worth remarking that a similar advantage could be obtained by measuring the intermediary system directly and not letting it fully thermalise in between measurements [50]. While there are clearly some differences due to the change in interaction, we find that introducing different waiting time distributions has a qualitatively identical effect in this case, i.e., the introduction of stochasticity allows us to significantly extend the range over which a thermometric advantage can be gained from the collisional thermometry scheme.

## 4. Conclusions

We have extended the framework of collisional quantum thermometry to include stochastic waiting time distributions (WTDs). We demonstrated that introducing a random WTD results in an advantage over the thermal Fisher information for a broader range of parameters, thus alleviating the need to precisely know the coupling strength with the environment. For a dephasing interaction between the collisional units and the intermediary system, we find that only classical correlations between the auxiliary units are established, and that, while these correlations appear to be a necessary ingredient to achieve the increased performance, there is not a clear one-to-one relation between the attained precision and degree of correlation.

## Data Availability

Not appliable.
